# The Tumor Microenvironment: A Pitch for Multiple Players

**DOI:** 10.3389/fonc.2013.00090

**Published:** 2013-04-17

**Authors:** Giovanna Schiavoni, Lucia Gabriele, Fabrizio Mattei

**Affiliations:** ^1^Department of Hematology, Oncology and Molecular Medicine, Istituto Superiore di SanitàRome, Italy

**Keywords:** tumor microenvironment, dendritic cells, macrophages, myeloid-derived suppressor cells, NK cells, T lymphocytes, cancer stem cells, solid tumors

## Abstract

The cancer microenvironment may be conceptually regarded as a pitch where the main players are resident and non-resident cellular components, each covering a defined role and interconnected by a complex network of soluble mediators. The crosstalk between these cells and the tumor cells within this environment crucially determines the fate of tumor progression. Immune cells that infiltrate the tumor bed are transported there by blood circulation and exert a variety of effects, either counteracting or favoring tumor outgrowth. Here, we review and discuss the multiple populations composing the tumor bed, with special focus on immune cells subsets that positively or negatively dictate neoplastic progression. In this scenario, the contribution of cancer stem cells within the tumor microenvironment will also be discussed. Finally, we illustrate recent advances on new integrated approaches to investigate the tumor microenvironment *in vitro*.

## The Tumor Microenvironment: Resident and Non-Resident Populations

Similarly to the majority of normal tissues, solid tumors are composed by two distinct compartments, the parenchyma and the stroma. The parenchyma is representative of cancer cells themselves, whereas all non-malignant cells and the other connective tissue elements belong to the stromal compartment (Dvorak et al., 2011). The stroma is a very heterogeneous milieu including various cell types and adhesion molecules, both contributing to the functional activity and structural support of the tumor microenvironment itself. Thus, the stromal and parenchymal regions may often be undistinguishable. For this reason, we can conveniently subdivide the cancer microenvironment into non-resident and resident components (Table 1).

**Table 1:** **Main structural and cellular components of the tumor microenvironment**.

Macrostructure	Subcomponents
**RESIDENT**	
Blood vessels	Endothelial cells
	Pericytes
Mesenchyma	Mesenchymal stem cells
	Mesenchymal cancer cells
	Fibroblasts
	Cancer-associated fibroblasts
	Cancer stem cells
Structural components	Adhesion molecules
	Cytokines
	Chemokines
**NON-RESIDENT**	
Infiltrating leukocytes	T lymphocytes
	B lymphocytes
	Monocytes
	Dendritic cells
	Macrophages
	Myeloid suppressor cells
	Natural killer cells
	Circulating stem cells
Tumor-derived cells	Metastatic cancer cells
	Cancer stem cells

Through this definition the resident component comprises cell populations and structural factors stably resident within the milieu of the stroma. In particular, endothelial cells and pericytes are resident cellular components surrounding and composing the blood vessels. Pericytes are structural cell components of blood vessels whose importance has been recently defined relatively to the cancer environment. Pericytes are commonly present in several organs and multicomponent cell structures (Fernandez-Klett et al., 2012; Iwasaki et al., 2012; Hellerbrand, 2013; Ren et al., 2013). Of interest, a functional role for these structures has been recently reported in the activation of innate immunity (Stark et al., 2013). In addition, pericytes can also display a functional property in cancer progression. This was shown in a study employing a transgenic mouse model expressing the viral thymidine kinase (tk) under control of the *NG2* promoter (NG2-tk mice) and transplanted with 4T1 breast cancer cells. In this work, Cooke et al. (2012) reported that treatment with Ganciclovir induced depletion of pericytic structures in tumors from NG2-tk mice but not in those from control mice, that was associated with markedly increased breast cancer progression and metastatic potential in mutant animals. The mesenchyma is another macrostructure belonging to the resident components, and is composed by several cellular types constantly present in this environment, such as fibroblasts, mesenchymal cells, and cancer stem cells (CSC). Other structural components such as adhesion molecules, cytokines, chemokines, and other biological compounds of functional relevance are all essential and confer to mesenchyma a time- and space-dependent functional role for the expansion of the tumor mass.

**Figure 1:**
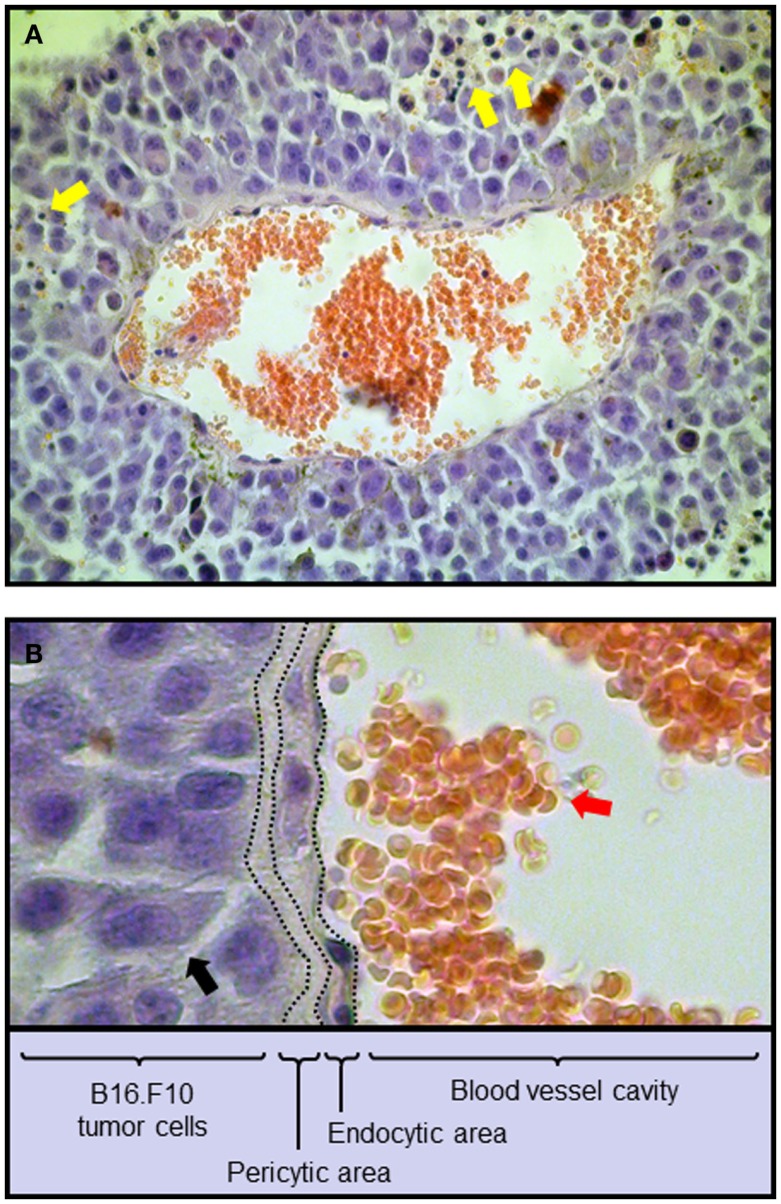
**Composition of the tumor microenvironment from a mouse melanoma tumor**. C57BL/6 mice were injected subcutaneously with 0.75 × 10^6^ B16.F10 melanoma cells. After 14 days tumors were excised and sections stained with the Hematoxylin/Eosin method. **(A)** 40× Magnification. Blood vessel with red blood cells (red circular cells) is shown. **(B)** Detail of the blood vessel depicted in **(A)** with the indication of the various functional structures linking blood vessel and tumor milieu. Yellow arrows, tumor-infiltrating leukocytes; red arrows, red blood cells; black arrows, melanoma cells.

Belonging to the non-resident constituent are different immune cell populations with the ability to infiltrate the cancer microenvironment by extravasation or through the help of blood vessels. In this scenario, resident and non-resident elements of the tumor microenvironment constantly interact and together represent a new forming, independent organ within the body (Table 1) (Dvorak et al., 2011). Hence, resident cancer cells produce selected chemokines that will set the composition of the infiltrating, non-resident fraction through the recruitment of leukocytes expressing specific chemokine receptors (reviewed in Toh et al., 2012; Viola et al., 2012). An example of cancer microenvironment composition is depicted in Figure 1 (Mattei et al., 2012), illustrating a section of a B16.F10 melanoma tumor grown in C57/Bl6 syngeneic mice. Hematoxylin/Eosin staining evidences the presence of infiltrating leukocytes surrounding the tumor mass (Figure 1A) as well as blood vessels and other structural components inside the tumor milieu (Figure 1B).

Recent studies indicate a role for microRNAs (miRs), pleiotropic regulators of gene expression, as critical components of the tumor microenvironment. Deregulation of miR expression at the tumor site has been reported to affect tumor progression and metastasis (Li et al., 2013). It has been evidenced that cancer cells are endowed with the ability to produce some classes of miRs, such as miR21 and miR29a (Fabbri, 2012; Fabbri et al., 2012). When produced by cancer cells, these little, non-coding, sequences of RNA were internalized by exosomes and secreted outside the cell, diffusing in the tumor milieu. By exploiting their expression of TLRs, such as TLR-7 (in mice) and TLR-8 (in humans), neighboring immune cells promptly captured these RNA sequences and internalized them via the exosomes. At this point, miR21 and miR29a exert their action by altering the transcriptional machinery of the cells (Fabbri, 2012; Fabbri et al., 2012). Of note, all immune cells expressing TLRs, such as DCs, are potentially exposed to this scenario. Therefore, miRs can be seen as integrated components of the tumor bed, acting as paracrine biological factors that contribute to the crosstalk between cancer and immune cells, together with the cytokine network generated therein, and orchestrating the fate of cancer progression.

## Immune Cells Infiltrating the Tumor Microenvironment: Role in Cancer Progression

Immune cell infiltration within a solid tumor is a naturally occurring event, when cells belonging to immune system enter within the tumor microenvironment by means of tumor-forming blood vessels and/or extravasation. This event can sometimes lead to blocking of cancer progression and thus limit or even prevent the generation of metastasis (Vesely et al., 2011). In general, this occurs when tumor progression is at early stages as a result of host immunosurveillance. At this point, if the organism is able to generate a prompt tumor-specific immune response, cancer progression is contrasted by immune forces and sometimes inhibited. In many cases, unfortunately, the host is unable to generate an effector immune response toward the tumor, due to the ability of the tumor to activate immunosuppressive mechanisms that circumvent or dampen immunity forces, thus leading to tumor escape from host immunosurveillance. In this scenario, the balance of immune populations endowed with effector vs. suppressive activities at the tumor site, is a critical parameter predicting cancer progression.

### Macrophages

Macrophages represent a significant portion of the tumor mass, and they certainly operate as fundamental actors in various types of solid cancers (Hao et al., 2012). These cells are generated from blood monocytes, which differentiate into two distinct macrophage types, schematically identified as M1 (or classically activated) and M2 (or alternatively activated) (Hao et al., 2012). In presence of LPS/IFN-γ, monocytes differentiate in the M1-polarized macrophages, characterized by the production of high levels of IL-1, IL-10, IL-23, TNF-alpha as well as CXCL10. Instead, M2-polarized cells are generated when monocytes differentiate in presence of IL-4, IL-10, and IL-13 (Martinez et al., 2009; Pollard, 2009). M1- and M2-polarized macrophages are endowed with opposite functional roles in terms of tumor suppression and immune stimulation. Indeed, whereas M1 cells, by virtue of their ability to elicit Mydd88/TLR pathways, enhance immune responses and restrain tumor progression, M2 macrophages switch-off the immune system and promote tumor development (Solinas et al., 2009). Moreover, human renal cell carcinoma has been reported to be frequently infiltrated with M2 tumor-associated macrophages (TAMs) promoting cancer progression. These TAMs produce high levels of IL-10 and CCL2, and display an elevated expression of 15-lipoxygenase-2 (15-LOX2) enzyme. Together, these factors allow infiltrating TAMs to promote inflammation, immunosuppression, and malignant progression of renal cancer carcinoma (Daurkin et al., 2011; Eruslanov et al., 2011). Furthermore, melanoma-infiltrating macrophages produce significant amount of adrenomedullin, which acts in an autocrine manner to promote their polarization toward the M2 phenotype. The generation of M2 macrophages enforce malignant progression and suppresses Cytotoxic T lymphocytes (CTL) activity as well as angiogenesis (Chen et al., 2011). Likewise, M2 macrophages were shown to play a crucial role in colon cancer progression. In a mouse model of colorectal cancer, TAMs were shown to play an indirect role by eliciting the recruitment of CCR6^+^T_REG_ lymphocytes inside the tumor mass, which then promote neoplastic progression by stimulating the secretion of CCL20 (Liu et al., 2011). In addition to sustaining tumor progression, M2 macrophages also promote angiogenesis. In this regard, mature F4/80^+^CD68^+^ alternatively activated M2 macrophages were shown to be endowed with a strong CTL-suppressive activity in a murine BW-Sp3 T cell lymphoma model (Van Ginderachter et al., 2006; Chen et al., 2011).

### T lymphocytes

Both preclinical and clinical studies suggest a strict correlation between the presence of tumor-infiltrating lymphocytes (TILs) with a favorable prognosis in a wide number of solid tumors (Galon et al., 2006; Fridman et al., 2012; Senovilla et al., 2012), although not all lymphocyte types are endowed with anti-tumor activity (Vesely et al., 2011). CTL play a relevant role in the process of tumor rejection. They are defined as CD8^+^ T cells producing massive amounts of IFN-γ and compounds necessary for their cytotoxic activity, such as granzyme B and perforin. The key role of CTL both in immunosurveillance against rising malignancies and in contrasting the metastatic expansion has been demonstrated in mouse models exploiting UV-induced skin cancers (Ward et al., 1990), chemically induced papilloma (Yusuf et al., 2008), and in the *ret* oncogene transgenic model of spontaneous melanoma (Eyles et al., 2010). High frequencies of circulating CD8^+^ T lymphocytes were detected in patients with metastatic melanoma, specific for Melan-A/MART-1, MAGE-10, and Ny-Eso-1, and CTL have also been found infiltrating melanoma metastases (Clark et al., 1989; Clemente et al., 1996; van Houdt et al., 2008; Fuertes et al., 2011). On this basis, adoptive transfer of CTL is therapeutically effective for mouse tumor models, although to a minor extent for cancer patients (Mempel and Bauer, 2009). These T cells, attracted by chemotactic stimuli secreted by the tumor mass, acquire the ability to migrate toward it. CTL do not require integrin to interact with neoplastic cells and are endowed with the ability to shift between components of tumor mass by means of ameboid locomotion (Weigelin et al., 2011). Time-lapse microscopy in a mouse melanoma model illustrated that melanoma-specific CTL effectively traffic to the melanoma site, where they engage contact with the B16.F10 cells via TCR/MHC-peptide interactions (Weigelin et al., 2011). Subsequently, tumor cells undergo apoptotic cell death induced by cytotoxic activity of CTL. These forming apoptotic bodies are made available for tumor-infiltrating dendritic cells (DC) that are thus allowed to sustain the systemic tumor-specific immunosurveillance by migrating to tumor-draining lymph nodes and other distant lymphoid organs, such as spleen, in order to present the processed peptides to naïve T cells. Moreover, it is also widely documented that the presence of TILs, including CTLs, is correlated with a favorable prognosis for several types of cancers, such as colon, breast, lung, ovarian, and esophagus cancer, just to cite a few (Naito et al., 1998; Schumacher et al., 2001; Zhang et al., 2003; Sato et al., 2005; Alexe et al., 2007; Al-Shibli et al., 2008; Fridman et al., 2012). The killing efficiency of target tumor cells by CTL is dependent on several factors, such as the cytokine/chemokine patterns produced by the tumor microenvironment, the molecular plasticity of tumor cells to evade CTL-induced killing and the strength of TCR/MHC-peptide interactions. In this regard, melanoma cells have been shown to down-regulate the MHC-I surface expression, thus bypassing the interaction with CTL via TCR (Mempel and Bauer, 2009). Of note, some reports demonstrated that induction of co-stimulatory molecule expression in human melanoma cell surface led to stimulation of CTL activity by Natural killer (NK) cells (Tarazona et al., 2004).

A significant fraction of TILs is composed by the regulatory T (T_REG_) cells, endowed with potent suppressive activity that counteract anti-tumor effector responses and favor tumor escape and progression. T_REG_ cells were reported to infiltrate a wide range of mouse and human tumors, such as melanoma, lung adenocarcinoma, breast cancer, and gastrointestinal tumors (Quezada et al., 2011; Wang et al., 2012b). Elevated frequencies of T_REG_ cells in peripheral blood and at the tumor site of cancer patients correlate with poor prognosis and reduced survival. However the mechanisms driving T_REG_ cell expansion, accumulation, and migration to the tumor site are currently unknown. It is likely, that factors relevant within the tumor microenvironment, such as the presence of TGF-β and low antigen stimulation, may play a role in the induction of T_REG_ cells *in vivo* (Quezada et al., 2011). Through the secretion of inhibitory cytokines and the expression of surface markers, T_REG_ cells inhibit the effector function of most immune cells, including T and B cells, DC, macrophages, and NK cells (Wang et al., 2012b). FoxP3^+^T_REG_ cells within tumor burden express elevated levels of multiple suppressive receptors such as PD-1, CTLA-4, TIM-3, LAG-3, and GITR (Sakuishi et al., 2010; Park et al., 2012b). The identification of these receptors has gained interest for the development of targeted anti-tumor strategies aimed at selectively depleting T_REG_ cells at the tumor site (Menetrier-Caux et al., 2012a,b; Wang et al., 2012b). For example, simultaneous blockade of CTLA-4 and PD-1 was shown to reduce the frequencies of T_REG_ cells and to increase the numbers of effector TILs in mice bearing established B16.F10 melanoma, improving the efficacy of tumor vaccines (Curran et al., 2010). Similar encouraging results have been obtained with melanoma patients, suggesting that approaches aimed at combining T_REG_ cell depletion-based immunotherapy with current tumor treatment protocols may be promising strategies in clinical oncology (Hodi et al., 2010). However, since PD-1 and CTLA-4 are not uniquely expressed by T_REG_ cells, these results may be attributable to the blockade or re-activation of other T cell subsets (Badoual et al., 2013). In some cases T_REG_ cells may also contrast tumor progression. Indeed, T_REG_ lymphocyte infiltration has been associated with favorable prognosis in several types of solid cancers, such as ovarian, bladder, head/neck, and colorectal tumors (Badoual et al., 2009; Fridman et al., 2012). The mechanisms by which T_REG_ cells exert opposite function, depending on the tumor type, are still under investigation, although it is plausible to hypothesize that the phenotypical uniqueness of each cancer microenvironment may elicit the recruitment of different T_REG_ cell subsets (Fridman et al., 2012).

### Dendritic cells

The involvement of DC in neoplastic progression became increasingly evident in the past 10 years, and has been shown by different research groups (Preynat-Seauve et al., 2006; Shurin et al., 2006; Fuertes et al., 2011; Galluzzi et al., 2012). Indeed, DC have been shown to infiltrate different types of primary solid tumors. The crucial role of these cells in the process of cancer progression is dictated by their unique property of potent antigen presenting cells (APC) capable to prime naïve T lymphocytes. Several investigations indicate that DC can be endowed with capability to yield an active tumor-specific immune response that ultimately reflects in tumor rejection (Preynat-Seauve et al., 2006). It has been demonstrated that DC present tumor-specific peptides to activated T lymphocytes during melanoma progression in tumor-draining lymph nodes (Fuertes et al., 2011; Gerlini et al., 2012a,b). On the other hand, the cancer immunosuppressive environment may cause DC to develop functional impairments resulting in failure to activate T cells. (Chaux et al., 1997a,b; Vicari et al., 2002). In this respect, DC may be viewed as a double-edged sword at the tumor site affecting either positively or negatively the anti-tumor response, depending on the composition of the tumor microenvironment.

Tumor-infiltrating DC (TIDC) are present in different types of solid cancers, including colon lesions and epitheliomas (Michielsen et al., 2011, 2012), but the largest number of TIDC has been found in melanoma (Furumoto et al., 2004). These TIDC are composed by myeloid and to a lesser extent plasmacytoid DC (Mattei et al., 2012). The latter display an immature phenotype *in situ*, but retain the ability to mature into fully competent APC following dissociation from the tumor bulk, without the need of cytokine or bacterial product exposure (Preynat-Seauve et al., 2006). In this regard, dying tumor cells are thought to provide maturation signals. Therefore, DC spontaneously infiltrate melanoma and other types of solid cancers, and are potentially endowed with the capability to process a soluble tumor-associated antigen (Preynat-Seauve et al., 2006). These TIDC then migrate toward draining lymph nodes in order to activate both naïve CD4^+^ and CD8^+^ T lymphocytes (Fuertes et al., 2011; Gerlini et al., 2012a,b). Therefore, newly generated cytotoxic CD8^+^ T cells may further contribute to tumor rejection by migrating toward the tumor site. The extent of tumor infiltration by mature DC has been often correlated with favorable prognosis in a wide array of clinical cancers (Cox et al., 2005; Ladanyi et al., 2007; Park et al., 2012c). In a mouse model of melanoma, it was recently shown that host immunodeficiency results in poor tumor infiltration by effector immune cells, such as T cells and DC, and closely associates with melanoma progression (Mattei et al., 2012). In this model, melanoma phenotype was shown to be shaped directly by cells of the immune system through release of soluble factors within the tumor microenvironment (Businaro et al., 2013).

Recent work demonstrated the crucial role of CD8α^+^ DC in the natural mechanisms of cancer immunosurveillance through response to endogenous type I IFN and induction of CTL-mediated tumor rejection (Diamond et al., 2011; Fuertes et al., 2011). The importance of CD8α^+^ DCs in anticancer response stems in their unique ability to process and present cellular antigens, in a process known as cross-presentation (den Haan et al., 2000; Joffre et al., 2012). Cross-presentation is the ability of APC to process exogenous antigens and present the derived peptides to the MHC-I molecules, rather than conveying them to the classical MHC-II pathway. Cross-presentation allows APCs to present those antigens that are not expressed by the APC itself and is crucial for the generation of CTLs against intracellular pathogens or tumors of non-hematopoietic origin. This occurs through a process named cross-priming and requires optimal stimulation of the cross-presenting DC (referred to as DC licensing) by means of “danger signals,” such as those emitted from damaged cells, or by inflammation (Kurts et al., 2010). In fact, in the absence of an activation stimulus that “licenses” DC, antigen cross-presentation does not result in cross-priming, but rather in cross-tolerance. Of note, type I IFN have been shown to provide strong signal for CD8α^+^ DCs promoting CD8^+^ T cell cross-priming *in vivo* against antigens derived from dying tumor cells (Lorenzi et al., 2011). However, the extent of a tumor-specific immune response in cancer patients is a rare event. It has been hypothesized that the reasons why a patient fails rather than succeed to give an effective immune response toward the tumor are essentially three, all strictly related to the composition of the cancer microenvironment: (i) immunosuppressive cytokines such as IL-10, TGF-β, VEGF, or PGE2, interfering with the generation of immunity (Pardoll, 2003), (ii) tumor-secreted chemokines that sequester antigen-loaded DC, thus impeding them to migrate toward lymph nodes (Hirao et al., 2000), (iii) inadequate ability of TIDC to capture tumor antigen, due to poor apoptosis within cancer microenvironment (den Haan et al., 2000). A recent research of Chiba and co-workers evidenced that the transmembrane protein TIM-3 can act as a molecular switch by which TIDC regulate innate immune responses against tumors. Indeed, several tumorigenic and angiogenic factors, such as VEGF and IL-10, released by the tumor *in situ* induce TIM-3 expression on TIDC surface, resulting in the suppression of innate immune responses to nucleic acids by binding to the damage-associated molecular pattern molecule, HMGB1 (Chiba et al., 2012; Mattei and Schiavoni, 2013). These findings have revealed a novel strategy of tumor escape from host innate responses to nucleic acids involving TIDC and provided important insights for therapy design involving TIM-3 targeting.

Some interesting findings suggest that tumor-specific immune responses can also occur in certain structures distinct from secondary lymphoid organs, the so called Tertiary Lymphoid Structures. Originally discovered for their importance in autoimmunity diseases (Takemura et al., 2001), these singular structures have subsequently been identified in human lung cancers (Dieu-Nosjean et al., 2008) and more recently in melanoma metastases (Cipponi et al., 2012). Tertiary Lymphoid Structures exhibit a well defined organization, with mature DC and T cells adjacent to B cell follicles, thus suggesting that these structures may be a site where tumor-specific T cell activation occurs (Fridman et al., 2012).

### Natural killer cells

Natural killer cells are large granular lymphocytes acting by their cytotoxic capacity and massive cytokine production. NK cells share with macrophages the surface expression of CD16 (FcyRIII), but are diversified from them by expression of CD7, CD56, and CD57 (Milush et al., 2009; Yasuda et al., 2011; Senovilla et al., 2012) and by mechanisms of pathogen killing. Indeed, whereas macrophages kill target cells by phagocytosis, NK cells mediate target cell lysis by secretion of perforin- and granzyme B-containing granules (Pardo et al., 2009; Afonina et al., 2010). A recent report demonstrated that NK cells are endowed with a potent ability to secrete calcium ions, and that this function allow these cells to increase their killing ability (Schwarz et al., 2012). For their killing activity, NK cells cover an important role in immune responses against tumors. Another interesting function of NK cells is the so called “DC editing.” This term specifies the ability of activated NK cells to interact with autologous DC and kill those cells that are not fully mature. Through this process, NK cells contribute to maintain the reservoir of immunogenic DC by killing potentially tolerogenic DC thus optimizing effector anti-tumor responses (Moretta, 2002; Moretta et al., 2006; Morandi et al., 2012).

The functionality of NK cells is fundamental for contrasting the growth and metastatic process of several types of cancers. For example, several reports have elucidated the role of NK cells in killing cancer cells in murine models of melanoma, colon cancer, lung cancer, and breast cancer (Azogui et al., 1991; Pham-Nguyen et al., 1999; Velthuis et al., 2003; Carrega et al., 2009; Frings et al., 2011; Kim et al., 2011; Takeda et al., 2011; Vesely et al., 2011; Roberti et al., 2012a; Srivastava et al., 2012). There are several NK receptors, such as DNAM-1, CD155, CD16, CD69, NKp30, and NKp46, whose surface expression is fundamental for maintaining cancer immunosurveillance (Clausen et al., 2003; Garcia-Iglesias et al., 2009; Lakshmikanth et al., 2009; Chan et al., 2010; Levy et al., 2011; Pfeiffer et al., 2011; Gleason et al., 2012; Park et al., 2012a). In this regard, some research groups investigated the intratumoral phenotypic profile and functions of NK cells in primary human tumors of non-small cell lung carcinoma (NSCLC) by using the NK cell marker NKp46. The data showed that intratumoral NK cells from these patients display a deeply altered phenotype that strongly contributed to the NSCLC progression in these patients (Platonova et al., 2011). These results strengthen the key role of the cancer microenvironment and its composition, and identify NK cells as important predictive biomarkers of neoplastic disease progression. A recent study utilizing mouse tumor models elucidated the importance of NK cells in a vaccination strategy against lung cancer. A survivin-based vaccination, coupled to the use of novel form of the 4-1BBL co-stimulatory molecule as an adjuvant has been effective in completely suppressing 3LL lung carcinoma progression. The vaccine efficacy was correlated with potent killing responses of NK cells (Srivastava et al., 2012). Another report highlighted the role of NK cells resident in lung tissue during the generation of lung metastases. These findings have elucidated that IFN-γ production by these lung-resident NK cells markedly repressed the formation of metastases in an experimental mouse melanoma model (Takeda et al., 2011).

Defects in NK cell number or phenotype are events that dictate the fate of neoplastic diseases other than lung carcinoma. Several investigations defined the key role of NK cell receptors in melanoma. This is the case of DNAM-1 that interacts with the CD155 NK-specific receptor and promote the killing of melanoma metastasis and the generation of a “cytokine storm” that contributes to the killing activity (Chan et al., 2010). Many studies reported possible mechanisms by which melanoma cells escape killing activity of NK cells (Balsamo et al., 2012; Pietra et al., 2012; Wang et al., 2012a). Nevertheless, strategies aimed at promoting and sustain the melanoma-specific killing activity of NK cells are only at early stages or poorly effective. A recent report showed an active role of the chemotherapeutic drug dacarbazine, largely used for this type of neoplastic disease, in activating the expression of NK1G receptor on NK cell surface, thus restoring the killing activity of NK cells toward melanoma cells (Hervieu et al., 2012). Similar encouraging approaches have been recently started for breast cancer by using the chemotherapeutic agent Cetuximab. This drug was shown to be effective in promoting NK cell killing activity in high relapse rate, triple negative breast cancer patients. Indeed, Cetuximab restored IL-2/IL-15-mediated NK cell killing activity, thus markedly improving the outcome of these patients (Roberti et al., 2012b). Taken together, these data strongly support a key role of NK cells in tumor progression. Indeed, when activated, NK cells fight malignant cells inside the microenvironment by direct killing as well as by contrasting the generation of metastatic foci.

### Myeloid-derived suppressor cells

Cancer patients and tumor-bearing experimental mice undergo dramatic changes in their hematopoietic progeny. These modifications are mainly due to accumulation of myeloid cells, including myeloid-derived suppressor cells (MDSC) (Serafini et al., 2004). These cells represent a heterogeneous population, either of monocytic or granulocytic origin, generated by and released from the bone marrow in response to a wide array of stimuli (Solito et al., 2011). MDSC are characterized by the surface expression of CD11b and Gr-1 markers, and their common functional feature is the repression of the effector functions of T lymphocytes and NK cells (Serafini et al., 2004; Gabrilovich and Nagaraj, 2009). For their potential to compromise both innate and adaptive immunity, tumor-infiltrating MDSC critically control cancer progression. By using STAT6^−/−^ mice models transplanted with 4T1 cancer cells, Sinha et al. (2005) showed that MDSC render 4T1 mammary tumors poorly immunogenic by suppressing the activation of CD4^+^ and CD8^+^ T cells. The involvement of STAT signaling in the immunosuppressive activity of MDSC in cancer has been further evidenced in studies on human head and neck squamous cell carcinoma and breast cancer, suggesting a pivotal role of STAT3 and STAT1 pathways, respectively, for MDSC suppressive activity within cancer microenvironment (Hix et al., 2013; Vasquez-Dunddel et al., 2013). Other independent investigations demonstrated that mice injected with 4T1 cells and subsequently treated with cyclophosphamide displayed tumor progression and metastatic spread despite drug treatment. Strikingly, the microenvironment of these tumor-bearing mice displayed a very high number of infiltrating MDSC, and this explained the unwanted inefficacy of chemotherapeutic cure. In addition, CD4^+^ and CD8^+^ T infiltrating cells have been shown to promote and sustain this effect by releasing IFN-γ (Guo et al., 2012b). Because the main property of MDSC is to contrast the immunosurveillance mechanisms of CD8^+^ and CD4^+^ T lymphocytes, the balance between T cells and MDSC within the cancer microenvironment may be a crucial cornerstone in dictating the fate of the neoplastic disease. Another recent report employing transgenic mouse models evidences that NK cells can potentially render T cells resistant to the suppressor activity of MDSC, and this markedly contribute to the generation of an effective adoptive transfer therapy with HER-2/neu tumor-reactive T cells and activated non-T cells, including NK cells (Kmieciak et al., 2011). In the mouse model of 4T1 metastatic breast carcinoma, accumulation of CD11b^+^Gr-1^+^MDSCs within the tumor tissue has been associated with tumor progression and bone metastasis (Bunt et al., 2007; Danilin et al., 2012) and a recent report showed that MDSC recruitment is triggered by Macrophage Migration Inhibitory Factor, an inflammatory cytokine expressed by the tumor (Simpson et al., 2012). Likewise, marked tumor infiltration and systemic expansion of CD11b^+^Gr-1^+^MDSC was found to be associated with enhanced tumor growth and malignant phenotype in an immunocompromised IRF-8^−/−^ mouse model transplanted with B16.F10 melanoma (Mattei et al., 2012). Together, these data strongly suggest a close correlation between intratumoral MDSC expansion with tumor progression and metastatic process.

The immunosuppressive functions of MDSC have been studied in several models of neoplastic diseases. It appears that MDSC may mediate T cell suppression through cell–cell contact or, alternatively, through release of soluble mediators, such as nitric oxide, arginase-1, reactive oxygen species or suppressive cytokines (e.g., IL-10) (Gabrilovich et al., 2012; Kerkar and Restifo, 2012). Studies on B16.F10 tumor-bearing mice have demonstrated that CCL5, a chemokine largely produced by MDSC (Zhang et al., 2012), is required for intratumoral recruitment of T_REG_ cells and melanoma expansion (Schlecker et al., 2012). Moreover, MDSCs have been shown to skew the differentiation of CD4^+^ T cells into T_REG_ (Huang et al., 2006), to induce an M2 phenotype in macrophages and to impair DC function, suggesting the existence of multiple overlapping regulatory mechanisms that dampen anti-tumor effector responses (Gabrilovich et al., 2012). Finally, MDSC facilitate tumor growth by producing pro-angiogenic factors, such as VEGF-A (Finke et al., 2011). Overall, MDSC exert pro-tumorigenic activity both through direct mechanisms and by employing a myriad of immunosuppressive mechanisms at the tumor site that blunt effector T cell responses.

### Other tumor-infiltrating immune cell subsets: γδ T and NKT cells

Besides the traditional players of anti-tumor immunity, emerging evidences suggest that other subsets of immune cells infiltrate solid tumors and may also contribute in cancer growth control. Among these, γδ T cells represent a small part of the lymphocyte population that share, with all T cells, the surface expression of TCR. Unlike αβ T cells, γδ T lymphocytes express a TCR complex where CD3 is associated to a γ and δ chains (Wu et al., 1988). These lymphocytes were originally characterized as strong cytotoxic and IFN-γ-producing cells, thus making them prototypic anti-tumor mediators. Indeed, γδ T lymphocytes have been linked to cancer progression since the early 1990s, when an encouraging study demonstrated their infiltration in human skin tumors, even though no clear indications about their function was given (Miescher et al., 1990). About a decade later, by employing mice lacking γδ T cells, Girardi et al. (2001, 2003) observed that these mice were highly exposed to carcinogen-induced cutaneous tumors, thus directly demonstrating the protective role of these cells in skin tumors. In the murine B16 melanoma model, γδ T cells were shown to infiltrate tumor lesions soon after transplantation and to provide an early source of IFN-γ (Gao et al., 2003). Other recent examples demonstrating a protective role of γδ T cells have been provided by two significant reports suggesting the presence of γδ T lymphocytes as a favorable prognostic factor for human breast cancer and melanoma (Kabelitz et al., 2007; Cordova et al., 2012; Ma et al., 2012). On the other hand, studies in mouse tumor models have demonstrated that γδ T cells within the tumor microenvironment exert an inhibitory action on CTL and NK cytolytic activity through production of IL-10 and TGF-β, resulting in the induction of tumor-specific immune tolerance (Seo et al., 1999; Ke et al., 2003). Recently, by employing IL-17^−/−^ mice two independent groups reported a subset of IL-17-producing γδ T cells infiltrating murine tumors, although opposite results were obtained regarding the anti-tumor activity of these cells. Wakita et al. (2010) showed that IL-17 produced by tumor-infiltrating γδ T cells promotes tumor progression by inducing angiogenesis in mouse models of methylcholanthrene-induced transplantable fibrosarcoma, skin, and colon carcinoma. In contrast, Zitvogel’s group showed in several subcutaneous tumor lines that early cancer infiltration by IL-17-producing γδ T cells is required for optimal tumor colonization of IFN-γ-producing CD8^+^ T cells and therapeutic efficacy of anticancer chemotherapy, in a mechanism requiring IL-1R1 and IL-1β, thus implying a beneficial role of IL-17-producing γδ T cells in anti-tumor defense (Ma et al., 2011; Mattarollo et al., 2011). Thus, the role of tumor-infiltrating γδ T cells in cancer immunosurveillance is still controversial and a more detailed characterization of γδ T cells is necessary in a wider set of preclinical tumor models that takes into account the phenotype of functional γδ T cell subsets.

NKT cells have also been reported to infiltrate solid tumors. Initially identified in 1981 by Minato et al. (1981) as a population expressing both a TCR and NK cell markers, NKT lymphocytes are currently known as a subset of innate-like T cell that recognizes Ag presented by CD1d (Kawano et al., 1997; Godfrey et al., 2004; Van Kaer, 2007). The best known subset of CD1d-restricted NKT cells, referred to as type I or invariant NKT (iNKT) cells, is characterized by surface expression of defined variants of α and β chain of the TCR, such as those encoded by vα24, vα14, vβ11, and Jα281 gene segments (Cui et al., 1997; Kawano et al., 1997; Kronenberg, 2005). These cells are conserved between humans and mice and are implicated in many immunological processes, such as production of massive amounts of several cytokines, both pro-inflammatory (i.e., IL-2, IL-17, IFN-γ, and TNF-α) and regulatory (i.e., IL-4, IL-10, and IL-13), which reflect their capacity to differently regulate anti-tumor responses (Swann et al., 2004; Bendelac et al., 2007; Exley et al., 2011). Pioneering work by Cui et al. (1997) showed that mice deficient for vα14^+^ iNKT cells were unable to mediate IL-12-induced rejection of tumors and that lack of infiltration of vα14 iNKT cell in melanoma tissue resulted in cancer progression and metastatic expansion. Subsequently, it was shown that the defective tumor immunity observed in Jα18^-/-^ mice, selectively deficient for iNKT cells, against methylcholanthrene-induced sarcomas and melanoma lung metastasis could be restored by adoptive transfer of IFN-γ-producing iNKT cells from WT donor mice (Crowe et al., 2005). iNKT cells can be recruited to the tumor by inducing local expression of chemokines, such as CCL21 or CXCL16 (Turnquist et al., 2007; Kee et al., 2013) and be functionally activated by the alpha-galactosylceramide producing beneficial effects in a variety of solid tumors (Nakagawa et al., 2000; Osada et al., 2004; Nagato et al., 2012). iNKT cell infiltration in primary tumors has been correlated with a favorable outcome in patients with colorectal carcinoma (Tachibana et al., 2005), neuroblastoma (Metelitsa et al., 2004), and hepatocellular carcinoma (Guo et al., 2012a). On the other hand, iNKT cells have also been described to suppress tumor immunosurveillance based on their production of Th2 cytokines (Terabe et al., 2000; Exley et al., 2011). It has been proposed that the instauration of a protective or suppressive anti-tumoral activity of iNKT cells may depend on the cytokine pattern of the cancer microenvironment and on the action of suppressive populations, such as MDSC and TAMs (Smyth and Godfrey, 2000; Terabe et al., 2003; Liu et al., 2012). Other, less characterized, CD1d-reactive NKT cells that have non-V24 (V14) invariant or variable TCR (type II NKT) as well as some non-CD1d-reactive NKT cell subsets that share with the NK cells the surface expression of a number of NK-associated receptors, such as CD161 (NK1.1), CD16, and CD56, have been described (Godfrey et al., 2004; Bendelac et al., 2007; Metelitsa, 2011). Several reports have demonstrated that type II NKT cells can mediate suppression of tumor immunosurveillance in multiple mouse tumor models by counteracting iNKT cell activities (Terabe et al., 2005; Berzofsky and Terabe, 2008; Izhak et al., 2013). However, these type II NKT cells have not been characterized in tumor infiltrates yet, indicating the need of further exploration for possible relevance of these cells in the local control of tumor growth and metastatic process.

## Cancer Stem Cells in the Tumor Microenvironment: Resident or Non-Resident?

**Figure 2:**
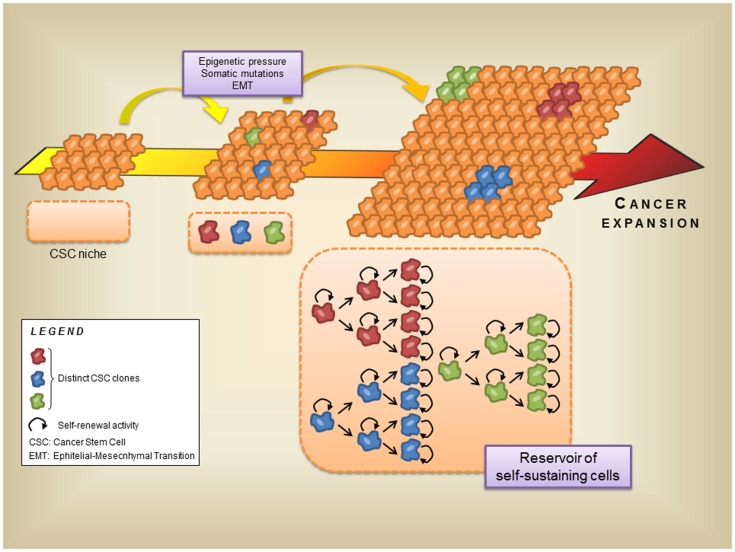
**Role of the cancer stem cells in the development of the tumor microenvironment**. Cancer progression is generated and sustained by several factors, such as epigenetic forces, somatic mutations, and EMT. On the other hand, these events lead to the generation of distinct cancer stem cell clones inside the cancer moiety, and enrich the cancer stem cell niche. During the late stages of tumor progression these stem cell clones sustain the cancer expansion with their self-renewal ability. Thus, the cancer stem cell niche can also be regarded as a reservoir of self-sustaining cells for the tumor microenvironment.

In the recent years, many reports proposed the definition of a new hierarchical model of cell organization, based on the discovery of a CSC. In this new model these tissue-specific, resident CSC acquire and retain the features of self-renewal, multilineage, differentiation, and tumor initiation both *in vitro* and *in vivo* (Boral and Nie, 2012). Importantly, CSC is the unique cell type of the tumor microenvironment able to initiate and maintain the formation of the environment itself and allow cancer progression. On the other hand, CSC are also equipped with the capability to colonize distant sites (Sengupta and Cancelas, 2010). Thus, in the context of solid tumors, CSC belong to both resident and non-resident compartments (Table 1). Nevertheless, despite a constantly increasing number of reports seem to stress a crucial role for CSC in carcinogenesis, there is so far no direct evidence that CSC are phenotypically tumorigenic cells significantly different from non-tumorigenic ones. All these reports postulate the Cancer Resident Cell (CRC) assumption on the simple observation that carcinogenesis is the result of cumulative mutations that develop during cancer progression. Genetic and epigenetic forces seem to work as main actors in this scenario. Hence, each mutated cell generates an independent, self-renewing clone of CRC, that will take part to the tumor mass contributing to its development (Gupta et al., 2009). As a consequence, it appears that the tumor microenvironment may theoretically be composed of an epigenetically heterogeneous population of CSC, representing self-renewing “stem clones” by which each tumor cell originates (Figure 2). CSC were first discovered in hematologic malignancies as the Acute Myeloid Leukemia tumor initiating cell, a primitive CD34^+^ hematopoietic cell susceptible to leukemic transformation (Bonnet and Dick, 1997), and successively identified in solid tumors, such as melanoma (Visvader and Lindeman, 2008; Shackleton and Quintana, 2010). The presence of melanoma CSC was discovered by using the immunocompromised mice NOD/SCID xenotransplanted with single human melanoma cell obtained from cancer biopsies (Quintana et al., 2008). Although the results demonstrated, for the first time in solid tumors, the existence of a single cell potentially endowed with the ability to generate a true tumor mass, no information was given about surface markers distinguishing the CSC phenotype (Quintana et al., 2008).

It has been recently elucidated that CSC can directly or indirectly interact with several immune cell populations, both *in vitro* and within the tumor microenvironment. These interactions are thought to influence the outcome of cancer progression. An interesting example comes from T_REG_ cells, endowed with the ability to produce and release TGF-β in the cancer microenvironment. TGF-β signaling and TGF-β-induced Endothelial-Mesenchymal Transition (EMT) are viewed as key orchestrators in the regulation of CSCs and T_REG_ participate in this process through producing TGF-β (Yu et al., 2012). Strikingly, a relevant research demonstrates that TGF-β and leukemia inhibitory factor (LIF) are responsible for the formation of neurospheres and the self-renewal ability of glioma-initiating cell (GICs) and that the effect of TGF-β is dependent on induction of LIF and JAK-STAT pathway (Penuelas et al., 2009). Moreover, T_REG_ cells, in concert with CSC, could affect the angiogenesis and VEGF-A level at the tumor site, especially when its microenvironment is hypoxic. This elicits vascularization extent and thus facilitates angiogenesis itself, which in turn allow CSC proliferation and maintenance inside the cancer microenvironment (Facciabene et al., 2011; Yu et al., 2012).

Effector T lymphocytes exert opposite effects to those observed with T_REG_ cells by induction of CTL capable to repress the expansion of CSC in ovarian cancer. This effect has been exploited to direct CTL to effectively killing ovarian CSC (OCSC) in a chemotherapeutic context. In this report, cell chimeras generated by fusion of DC and OCSC to specifically target the OCSC subpopulations were shown to activate T cells to express elevated levels of IFN-γ with potent killing activity of CD44^+^ OVCA cells (Weng et al., 2011). The NK have also been reported to affect CSC activities. It has been demonstrated that NK cells are endowed with the ability to kill human melanoma stem cells with high levels of the CD133 marker (Pietra et al., 2009). In this study, the authors were able to show that CD133-positive melanoma CSC, isolated from a FO-1 melanoma cell line by cell enrichment, were effectively killed by activated NK cells. Moreover, myeloid-derived cells such as DC may be effective in targeting Glioma stem cells (GSC), leading to the complete eradication of this malignant primary brain tumor (Ji et al., 2013). Here, the authors prepared a tumor vaccine by loading DC with U251 human GSC lysates and showed that these antigen-loaded DC are endowed with the ability to induce tumor-specific CTLs that killed glioma cells *in vitro*. TAM have also been shown to modulate the tumorigenic and angiogenic potential of CSC within tumor transplanted mouse model (Jinushi et al., 2011). A recent study reported that inhibition of TAM by targeting either the myeloid cell receptors CSF1R or CCR2 decreases the number of tumor initiating cells in pancreatic tumors (Mitchem et al., 2012). Thus, inhibiting macrophages function can lead to cancer eradication via diminishing the presence of CSC inside the tumor microenvironment. Finally, little is known about the interactions between MDSC and CSC. However, since MDSC are pivotal for the generation and maintenance of an aggressive cancer microenvironment, it has been recently hypothesized that these cells may also act as a distinct tumor niche whose main function is the maintenance of self-renewal ability of the niche itself, thus functionally resembling to CSC (Ye et al., 2010).

Overall, CSC are an important and integrated component of the cancer microenvironment. Through their self-renewal ability, CSC also function by tightly interacting with the other resident and non-resident component of the environment, thus crucially controlling cancer progression and maintaining the tumor mass.

## New Approaches to Study the Cancer Microenvironment

Exploiting complex experimental systems such as confocal microscopy, electron microscopy or two photon microscopy allow oncoimmunologists to “capture” suggestive images finely representing the cell–cell interactions occurring in biological systems. However, the existent traditional experimental protocols do not allow monitoring and visualizing in real time the interactions between immune and tumor cells occurring inside the cancer microenvironment.

Over the past decade, innovative approaches devoted to the reproduction of biological systems at the microscale, such as microfluidic platforms, have gained interest for the study of one or multiple biological systems. The first pioneering studies on biosensors performed in 1990s to investigate on the kinetics between the antigen-antibody interactions gave a decisive boost on this research area (Karlsson et al., 1991). This subsequently led to the engineering of microfluidic systems to investigate on biological fluids (Wilding et al., 1994) and, subsequently, on cell systems (Li and Harrison, 1997). Since 2004 microfluidic approaches have been employed to investigate on cells belonging to immune system to investigate migratory and morphological parameters in response to external stimuli (Lin et al., 2004; Wong et al., 2008; Butler et al., 2010; Li et al., 2011). In addition, a magnetic microfluidic chip has been developed to isolate circulating human colon cancer cells (Choi et al., 2010; Xia et al., 2011) or to study chemoattractant properties of tumor cells (Goerge et al., 2007). Other studies have exploited cell-on-chip based platforms to investigate the susceptibility of tumor cells to chemotherapeutic drugs (Liu et al., 2007; Siyan et al., 2009; Kim et al., 2012). Thus, the extreme versatility and the high customization potential of microfluidic-based technologies have open promising perspectives on the use of these approaches for cancer biology studies (Wlodkowic and Cooper, 2010; Wlodkowic and Darzynkiewicz, 2010).

In this context, the reconstitution on-chip of the cancer microenvironment may allow to elucidate the fine mechanisms that mechanically regulate the interactions of tumor cells with other resident and non-resident components, such as the immune cells, as well as the mechanisms driving the differentiation and metastatic mobilization. Recently, a simple cell-on-chip approach has been developed to investigate the crosstalk between two complex biological systems, such as immune system and cancer (Businaro et al., 2013). By using a microfluidic platform consisting of three wide, parallel chambers interconnected via an array of short and narrow capillary migration channels, Businaro and Colleagues were able to visualize and follow, “under the microscope,” the interactions between the immune and cancer cells. The goal of the study was to investigate the role of IRF-8 immune deficiency on immune response to melanoma. Thus, splenic immune cells from IRF-8^−/−^ mice or WT controls were allowed to interact with B16.F10 melanoma cells into the microfluidic structure. The results evidenced a marked inability of IRF-8^−/−^ immune cells to migrate toward and interact with melanoma cells with respect to WT cells. In turn, melanoma cells acquired a more invasive behavior in the presence of immunodeficient cells, indicating a crosstalk between cancer and immune cells shaping the phenotype of tumor cells. Notably, these results are fully compatible with the findings reported *in vivo* by the same group, suggesting the reliability of the system (Mattei et al., 2012). The observations reported in this study are difficult to obtain with the standard well plate culture experiments, but are easily available with the use of a microfluidic platform. Thus, customized microfluidic platform may be potentially helpful to study, follow, and mimic the plethora of events occurring inside the cancer microenvironment. In addition they can also be utilized as helpful tools in preclinical and clinical investigations.

## Concluding Remarks

Immune cell infiltration within the tumor microenvironment is a crucial requisite for a successful and prompt eradication of the primary tumor itself. Each cell type herein supports the maintenance of the microenvironment itself both functionally and biochemically, by orchestrating cell–cell interactions and secreting a plethora of chemokines and cytokines. This leads to a fine tuning of the timing and modality of immune response, as well as to an appropriate modulation and maintenance of angiogenic processes. The final effect of such a strictly related phenomena may either be the generation of a tumor-specific immune response or tumor escape (Table 2).

**Table 2:** **Immune cells infiltrating the tumor microenvironment and their role in tumor progression**.

Cell type	Representative infiltrating population	Outcome	Main features of infiltrating cells inside the tumor microenvironment
**Macrophages**	M1	TR	Activation of immune responses by MyDD88/TLR pathways
	M2	TP	Promotion of angiogenesis; suppression of CTL function; recruitment of CCR6^+^T_REG_; positive modulation of the tumorigenic and angiogenic potential of CSC
**T lymphocytes**	CTL	TR	Specific tumor cell killing activity
	T_REG_	TP/TR	TP: functional suppression of CTL, DC, NK cells, and macrophages.
			TR: correlation with good prognosis in some solid tumors, hypothetically due to lacking of suppressor activity and other unidentified activities
	γδ T cells	TP/TR	TP: inhibition of CTL and NK cell activity; promotion of angiogenesis
			TR: cytotoxic activity, IFN-γ production
**Dendritic cells**	CD8α^+^ DC	TR	Processing and presentation of soluble tumor-associated antigens; Type I IFN-dependent CD8^+^ T cell cross-priming against antigens released from dying tumor cells
	Plasmacytoid DC	TR	Processing and presentation of soluble tumor associated antigens
	TIM-3^+^ DC	TP	Suppression of HMGB1-dependent innate immune responses
**NK cells**	NKp46^+^	TR	Specific tumor cell killing activity by secretion of perforin and granzyme B-containing granules as well as release of calcium ions; DC editing; killing activity against CSC
	NKp30^+^	
	DNAM-1^+^	
	CD69^+^	
	CD155^+^	
**NKT cells**	NK receptors	TP/TR	TP: CD1d-restricted cytotoxic activity; IFN-γ production; APC stimulation
	TCR-α chain variants	
	CD1d-restricted		TR: Th2 cytokine production
	CD57^+^	
**Myeloid-derived suppressor cells**	CD11b^+^Gr-1^+^	TP	Repression of the effector function of T lymphocytes and NK cells; highly present in late stages of tumor progression; promotion of T_REG_ functions; promotion and sustainment of angiogenesis; present in elevated number in highly aggressive microenvironments
**Cancer stem cells**	CD34^+^ CD133^+^	TP	Self-renewal function; tumor initiating activity; promotion and sustainment of angiogenesis; tumor resistance; sustainment of the tumor mass

*TP, tumor progression; TR, tumor regression*.

Thus, a better knowledge of the crosstalk between cancer cells and immune system cells occurring inside the tumor bed is of interest for further improving cancer therapies. In this respect, great benefit for anticancer research and clinical practice may arise from exploiting new versatile biotechnologies, such as microfluidic cell-on-chip platforms.

## Conflict of Interest Statement

The authors declare that the research was conducted in the absence of any commercial or financial relationships that could be construed as a potential conflict of interest.
